# Medical Society of Tennessee

**Published:** 1845-06

**Authors:** 


### THE WESTERN JOURNAL.

New Series.—Vol. III.—No. VI.

LOUISVILLE, JUNE 1, 1845.

### MEDICAL SOCIETY OF TENNESSEE.

From a correspondent at Nashville, we have received the following particulars of the late meeting of the Medical Society of Tennessee, in that city.

The meeting was held on the first Wednesday in May, and seems to have called out an unusual number of addresses and communications. Several of the cases were ordered by the Society to be transmitted to the Western Journal for publication, and we have to request the Secretary to forward them at his earliest leisure.

Dr. Winston, orator of the meeting, delivered, says our correspondent, a very handsome address on the contributions to medical science by American physicians. He defended the character of the profession in our country, and insisted on their claims with much ability.

The President of the Society, Dr. Buchanan, read an essay "*on the difficulty of acquiring accurate knowledge in practical medicine,*" in which he attempted to show, that the French mode of cultivating medical science, *if the true mode,* is not the *only* true mode. This, he contended, is manifest from the fact that many diseases were treated as successfully before, as they have been since the development of the peculiar doctrines of the numerical school. Hence, he would not hastily throw aside the old method of observation of symptoms, and of the effect of remedies in modifying them, even although we should be ignorant of the organic lesions upon which they depend, but, on the contrary, would take either one of the two roads to the Temple of Truth, as it might seem most eligible at the time and under the circumstances.

Dr. Robards read an excellent paper on the Epidemic Erysipelas of Maury county, which proved so extensively fatal there last spring and summer.

Resolutions were passed by the Society to erect a monument to the memory of the late Dr. Samuel Hogg. The funds are to be raised by subscription, and we doubt not that every physician in the State will feel a pleasure in contributing his part towards this worthy object.

The Society resolved to maintain its organization, and for the purpose of enhancing the interest of its next annual meeting, offer a premium of $50 for the best essay on Scrofula.

The friend to whom we are indebted for these items of news expresses a wish, that the address of Dr. Winston, and the other papers brought out by the meeting of the Society, were in the hands of the editors of this Journal. We join with him in this wish, and hereby express the hope that he will take measures to have them sent to us at an early day. The Society, he says, is not able to publish any of the long essays, in all of which, he supposes, there are suggestions worthy of being given to the profession.

We would here remark, that we have never received some matters ordered to be communicated to this Journal by the Society at its meeting a year ago, and that they would still be very acceptable.

